# Evaluation of colonoscopy in the diagnosis and treatment of rectal carcinoid tumors with diameter less than 1 cm in 21 patients

**DOI:** 10.3892/ol.2013.1214

**Published:** 2013-02-27

**Authors:** JING LIU, ZH-QIANG WANG, ZI-QI ZHANG, XIAO CHEN, YU ZHANG

**Affiliations:** Department of Endoscopy of the South Building, The PLA General Hospital, Beijing 100853, P.R. China

**Keywords:** colonoscopy, endoscopic ultrasonograph, carcinoid tumor, endoscopic mucosal resection, pathology

## Abstract

The aim of this study was to evaluate colonoscopy in the diagnosis and treatment of rectal carcinoid tumors with diameter <1 cm. Elevated lesions with normal mucosal appearance under colonoscopy were identified. Endoscopic ultrasound (EUS) was performed in 16 patients. Lesions diagnosed as rectal carcinoid tumors were resected by endoscopic mucosal resection (EMR). The diagnosis of specimens by EMR was confirmed by pathological examination. Immunohistochemical staining was undertaken and follow-up data were collected. Twenty-two lesions were found among the 21 cases. The majority of these were located within 10 cm of the anal opening. Twenty two cases with rectal carcinoids were diagnosed by EUS under colonoscopy and all cases were verified by pathological examination. The resection rate was 95.5% (21/22). Of the lesions, six were mucosal and 10 were submucosal. Immunohistochemistry was undertaken for carcinoid tumors. Histological patterns of rectal carcinoids revealed solid nests or trabecular patterns. Eleven cases were synaptophysin (SYN)-positive, 8 cases were neurone-specific enolase (NSE)-positive and 5 cases were chromogranin A (CgA)-positive. Colonoscopy combined with EUS is effective in the diagnosis and determination of small rectal carcinoids. Endoscopic treatment is effective for small-sized tumors. Pathology and immunohistochemistry remain the diagnostic gold standard.

## Introduction

Rectal carcinoids are low-grade malignancies, which are slow-growing and usually become symptomatic late in the course of the disease (1,2). The incidence of rectal carcinoids is rising: In the United States, the age-adjusted incidence has increased by 800–1000% in the last 35 years. The incidence of rectal carcinoids is the highest among gastrointestinal carcinoid tumors (3). The incidences of carcinoids of the rectum, stomach and small bowels have also multiplied (4). The reasons for these epidemiological changes are not yet understood. Screening colonoscopy, in addition to decreasing colorectal adenocarcinoma mortality, is useful in diagnosing carcinoid tumors at an earlier stage and in decreaing mortality form malignant colorectal carcinoid tumors (5,6).

Small carcinoid tumors measuring <1 cm in diameter may therefore be managed endoscopically and preoperative assessment with endoscopic miniprobe ultrasonography (EUS) with out recurrence or spread (7,8). Various methods for complete endoscopic resection of rectal carcinoid tumors have been reported, such as endoscopic submucosal dissection (ESD) or conventional endoscopic mucosal resection (EMR) (9,10). Increased surgical time and complication risks with ESD remain problematic (11). The selection of endoscopic treatment should be made after taking factors, such as cost-effectiveness, expertise and experience into careful consideration (12). In this study, we review and summarize the endoscopic and pathological features of small rectal carcinoid tumors (≤1 cm in diameter) of twenty-one cases. We also evaluate the safety and efficacy of complete resection of small rectal carcinoid tumors using EMR and endoscopic submucosal resection with cap aspiration technique (ESMR-C).

## Patients and methods

### Patient cases

A series of 22 rectal carcinoid tumors in 21 patients (age range, 37 to 78 years; 2 females and 19 males) were treated at The PLA General Hospital, Beijing, China, between June 1995 and March 2010. All clinical data and pathological records were reviewed. All cases were detected by colonoscopy without clinical symptoms. The study was approved by the Ethics Committee of The PLA General Hospital, Beijing, China. Written informed consent was obtained from the patients.

### Devices

CF-H260AL colonoscopy (Olympus, Tokyo, Japan) and micro ultrasound probe UM2R and UM3R (Olympus) were used with frequencies of 12MHZ and 20MHZ. The aerodynamic endoscopic ligation device MD-48709 was employed (Olympus).

### Methods

Under colonoscopy, the rectal carcinoid tumors were identified to have smooth surfaces and normal mucosa. In 16 cases, EUS was performed using the degassing and water filling method to observe the size of the lesion and the shape of the border, the internal echo intensity, sources and the relationship between the lesion and the surrounding wall. All patients were followed-up after 2 months, 6 months and 1 year by colonoscopy, EUS and local biopsy.

## Results

### Lesion location, size and shape

**Figure 1:**
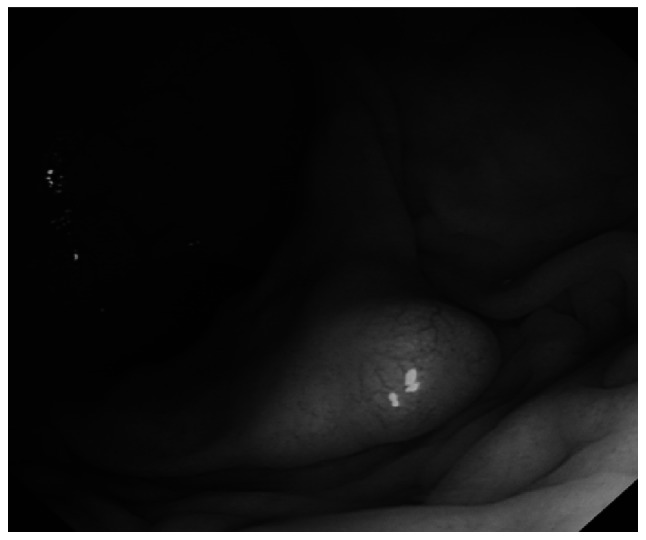
Appearance of rectal carcinoid under colonoscopy, the tumor was hemispherical bulgeshape with smooth surface and normal mucosa.

Only 1 patient had a lesion that was 15 cm from the anus, while the rest were within the range of 3–10 cm from the anus. Two lesions were found 6 cm away, and 1 case was 8 cm away from the anus. The size of the lesions ranged from 0.3 to 1.0 cm. Surface erosion was found in 1 case, while the remaining cases were smooth. Lesions were yellow in 7 cases and slightly red in 3 cases. The remaining lesions were similar in colour to the surrounding mucosa. The tumor shapes were hemispherical bulges in 13 cases and flat-shaped bulges in 9 cases. Upon being touched with bopsy forceps, a sliding movement was observed in 8 cases (Fig. 1).

### EUS results

**Figure 2:**
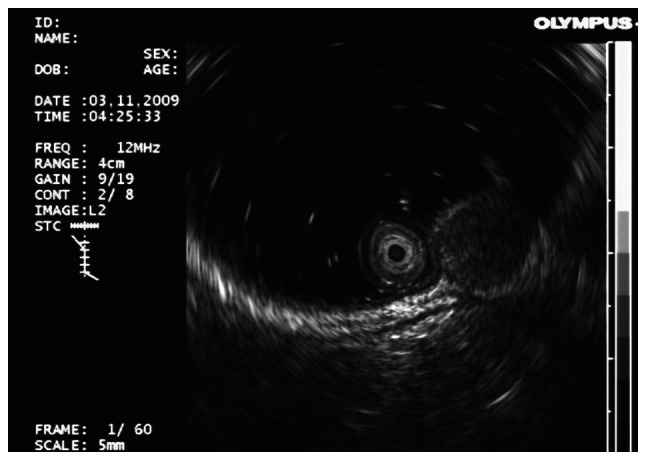
Appearance of rectal carcinoid under endoscopic ultrasound demonstrates hypoechoic nodules located in the submucosa with clear boundaries.

Among the 16 cases of rectal carcinoid lesions, 12 were hypoechoic nodules and 4 were isoechoic nodules. Six lesions were located in the mucosa and 10 lesions were located in the submucosa. Sixteen lesions showed clear boundaries (Fig. 2).

### Treatment and follow-up results

**Figure 3:**
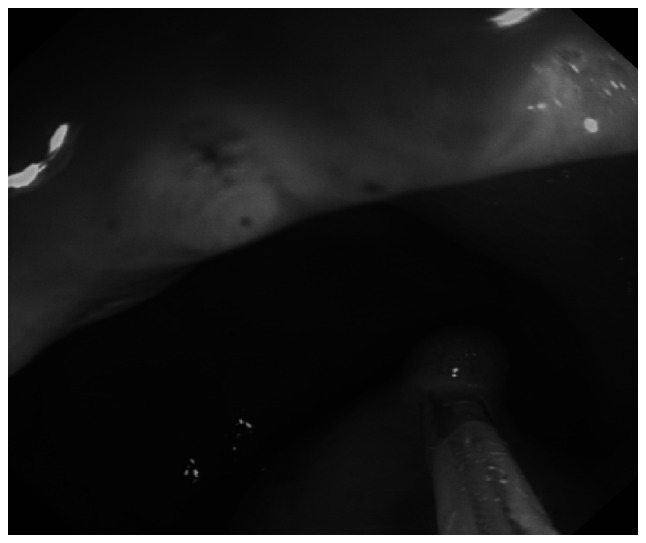
A snare resection was performed by electrosurgical current after submucosal injection solution.

**Figure 4:**
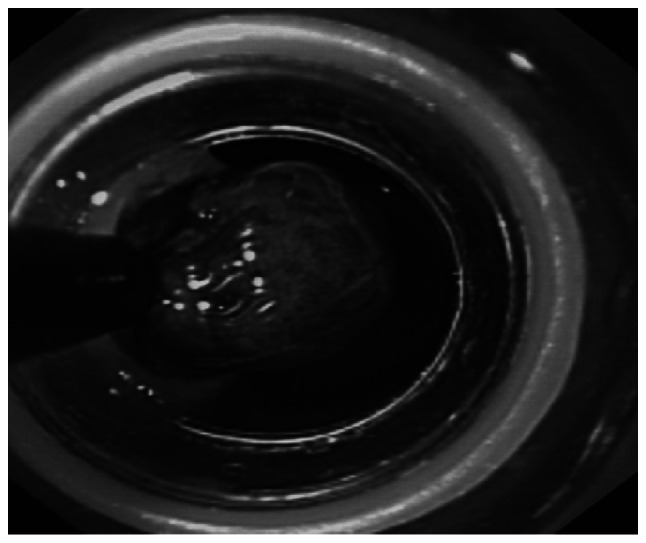
The lesion was immobilized with suction using a endoscopic ligation device.

Different treatments were selected according to the size and shape of the lesion. For lesions with a diameter of ∼0.5 cm and a semisphere shape, a snare resection was performed by use of an electrosurgical current following a submucosal injection of physiological saline solution around the lesion to lift it off the muscle layer (Fig. 3). For flat lesions or lesions with a diameter of >0.5 cm, prior to the snare resection, the lesion was immobilized with suction using an endoscopic ligation device (Olympus) (Fig. 4). For 1 case with a minor lesion of 0.2 cm, the lesion was clamped using biopsy forceps and then resected by argon plasma coagulation (APC) using a VIO 300D electrosurgical system (ERBE, Tuebingen, Germany). The specimens were pathologically examined and immunohistochemistry was conducted for some specimens.

**Figure 5:**
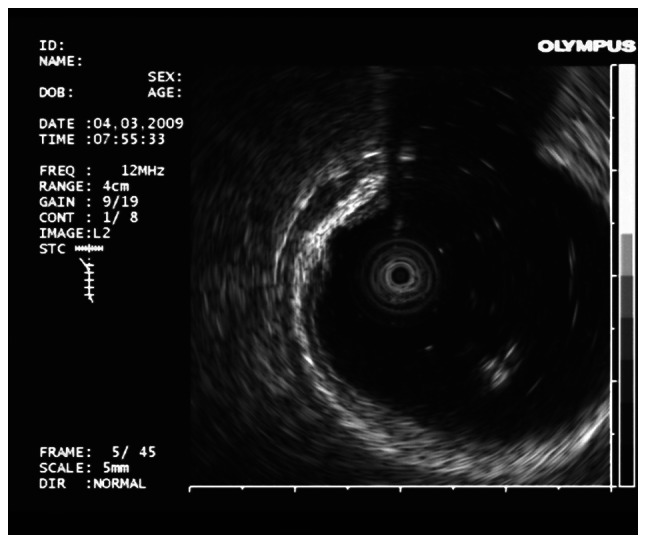
Ultrasonographic examinations showed no local carcinoma after excision.

**Figure 6:**
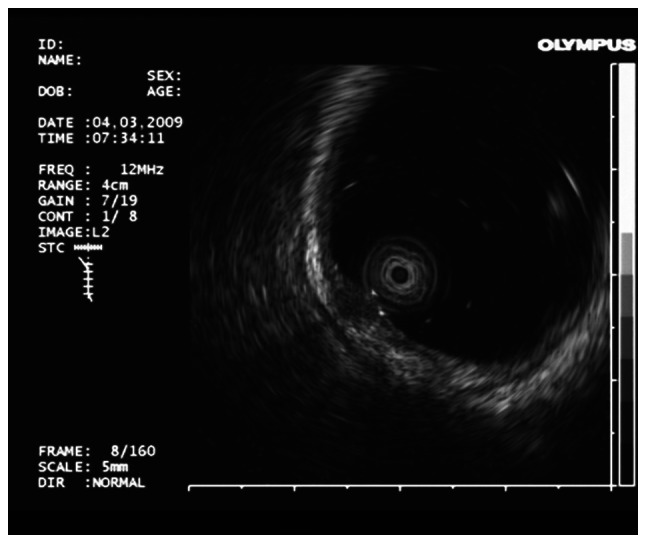
Ultrasonographic examinations still found carcinoid tissue in the in the edge after 2 months.

Following excision of 18 lesions, pathological examination revealed a clear cutting edge and base. After 2 months, the follow-up found no carcinoid tumor tissue. Three cases reported carcinoid tissue in the margin. After 2 months, the ultrasonographic and pathological examinations showed no local carcinoma (Fig. 5). One case reported carcinoid tissue in the margin. After 2 months, the ultrasonographic and pathological examinations still found carcinoid tissue in the margin and surgical operations were undertaken (Fig. 6).

### Pathological results

**Figure 7:**
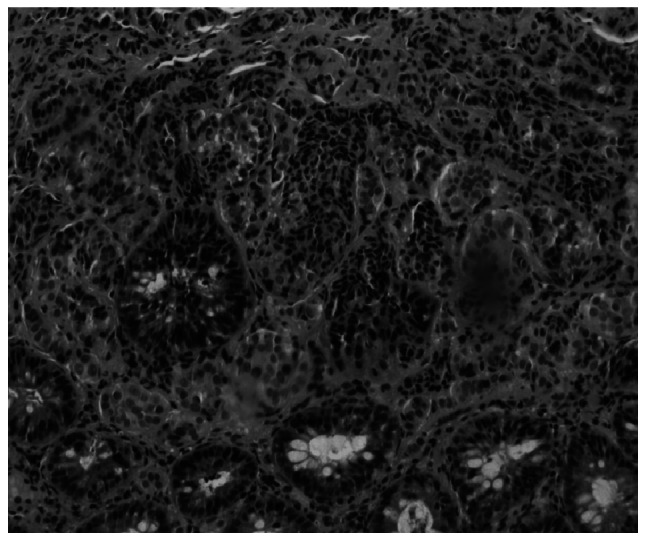
Pathological results of rectal carcinoid. Pathological results of rectal carcinoid with solid nest-like, island-like nodules and abundant capillaries. Heamatoxylin and eosin staining. Magnification, ×40.

**Figure 8:**
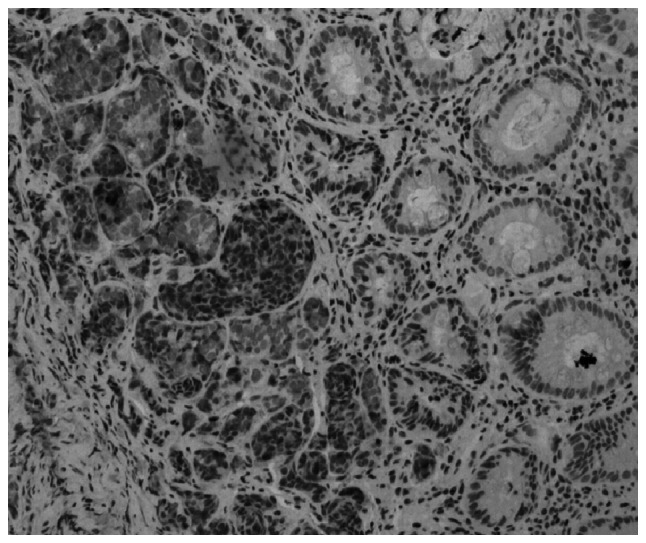
Synaptophysin was positive in rectal carcinoid tissues. SP, ×200.

**Figure 9:**
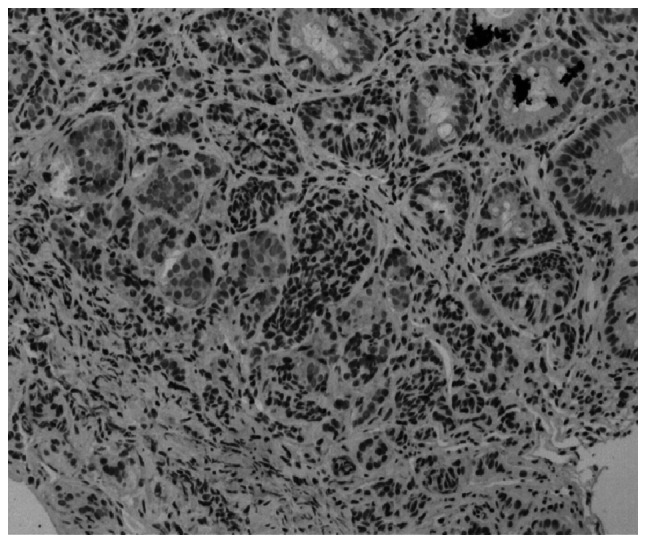
Neuron-specific enolase was positive in rectal carcinoid. SP, ×200.

Pathological examination revealed chronic inflammation in the surface of the rectal mucosa. Microscopically, the tumor cells showed solid- or cord-like appearances with round or polygonal shapes and small nuclei. The majority of the tumor cells were confined to the mucosa or the submucosa (Fig. 7). Eleven samples of 21 cases received immunohistochemistry evaluation. Among these, vesicle synaptophysin (SYN) was positive in 11 cases (Fig. 8), neuron-specific enolase (NSE) was positive in 9 cases (Fig. 9), chromogranin (CgA) was positive in 5 cases and CK was positive in 2 cases. Ki-67 and CEA were negative.

## Discussion

Rectal carcinoid tumors originate from the Kulchisty cells (or chromaffin cells) in the deep intestinal crypt. These amine precursor uptake decarboxylase (APUD) cells, known as neuroendocrine tumors, are low invasive with limited infiltrating growth. A single tumor is common, while multiple tumors account for only 2 to 4.5% (13)t. Compared with other tumors, lymphatic and hematogenous metastasis is less. Among our cases, only 1 case was multiple. It has been reported that multiple tumors are only 0.5 cm away from each other, probably due to submucosal lymphatic metastasis, rather than multiple germinal generation (13).

In general, rectal carcinoid tumors are more common in the front or lateral wall of the rectum, usually 3 to 8 cm away from the anus. The early small nodules in the lamina propria gradually develop into the submucosal and muscle layer. Colonoscopy has revealed a flat round nodule with a clear border and a smooth surface, coated with the normal mucosa, presenting a white or light yellow color and movability with biopsy clamp touch. As the diameter of early lesions is <1 cm, they are initially diagnosed as submucosal tumors.

As the shape of the rectal carcinoid tumor resembles sessile polyps, certain endoscopists may regard carcinoid tumors <0.5 cm as hyperplastic polyps and ignore them, thus delaying the treatment. Although occasionally biopsy for pathological examination is conducted, the tumor tissue is difficult to biopsy since it is located in the submucosa (14). It is also difficult to correctly determine the size, the intestinal origin and the histological features of the rectal carcinoid tumor with common colonoscopy. Rectal endoscopic ultra-sound is the most accurate method to determine the depth of tumor invasion. With high ultrasound frequency (12–20 MHz), EUS is able to clearly show the five-layer structure of the digestive tract wall, determine the source of the tumor and its relationship with the rectum wall, and the nature of the lesion, which aids in making accurate judgements in order to determine the treatment. EUS is the best for diagnosis of tumors of gastrointestinal mucosa and submucosa. EUS boasts high accuracy for the identification of benign and malignant rectal carcinoid and other submucosal tumors, as well as extraintestinal oppressive lesions (15,16).

In our study, we used this examination technique in 16 cases of rectal carcinoid lesions, which all showed hypoechoic or isoechoic nodules with clear boundaries. Six lesions were located in the mucosa and 10 lesions were located in the submucosa. According to the literature, leiomyoma from the muscularis mucosa also present with sonographic features of hypoechoic nodules with clear boundary and uniform internal echo. The sonographic features of the rectal leiomyoma and the rectal carcinoid tumor require further research. No leiomyoma of the rectum has been confirmed pathologically in our department, due to the much lower incidence than rectal carcinoid tumors under endoscopy. Therefore, priority EUS should be given to rectal carcinoid displaying EUS features, such as hypoechoic or isoechoic nodule with clear boundaries located in the mucosa or submucosa (17).

Tumor size is closely correlated with the degree of malignancy. A diameter of 2.0 cm is the key indicator to determine the level of malignancy of the rectal carcinoid tumor (18). The rate of metastasis of tumors with diameters <1.0 cm is <2%. For 2/3 of rectal carcinoid tumor, the diameter is <1.0 cm. The diameters of the lesions were between 0.3 and 1.0 cm with clear borders. We believe that endoscopic treatment should be first considered. The resection should include the normal mucosa 0.5 cm away from the border of the tumor and the depth should reach the muscle layer. Pathological results confirmed the carcinoid tumor, and that the edge and base were intact without residue. For hemispherical elevated lesions <0.5 cm in diameter, direct snare electrocoagulation resection was performed after submucosal injection of saline. For lesions with diameter >0.5 cm or flat lesions, with the application of endoscopic air-driven ligator (transparent cap attached to the tip of the intestinal endoscope) and the formation of polypoid bulge, the electrical incision ring was used to remove the lesions (EMR). To avoid residual tumor, EMR is more effective (19). Electrocautery may be used to stop bleeding after EMR. For residual tumor confirmed by pathology, due to the thermal conductive effects, the residual tumor tissue may be in coagulation and necrosis. Close follow-up will be required instead of expanding the surgery. EUS and endoscopic biopsy are needed after 1 to 2 months. This approach has been successfully utilized to treat 21 cases of rectal carcinoid tumors with a diameter of <1 cm in our department. No complications, such as bleeding or perforation, occurred. Six months to 1 year follow-up showed no recurrence.

Immunohistochemical examination is essential for determining the diagnosis of carcinoid tumor. All tissues in solid nest-like, island-like nodules, beam-like, banded or rose-like structure or with abundant capillaries or sinusoids should undertake the routine neuroendocrine markers immunohistochemical examination (20–22). Among known neuroendocrine markers, SYN, NSE and CgA are relatively specific, and SYN is relatively sensitive in rectal carcinoids. Expression of Ki-67 is important for assessment of tumor cell proliferation activity and prognosis prediction (23). In our study, 11 cases undertook routine immunohistochemistry examination, of which 11 were positive for Syn, 9 were CgA positive and 5 were positive for NSE, while CEA was negative in all cases. The expression rate of Ki-67 in rectal carcinoid tumors is low. In this study, the expression rate of Ki-67 in tumors lesions <1 cm was <5% or negative. These were attributed to the low degree of malignancy of rectal carcinoid tumors.

In this study, we showed that colonoscopy combined with ultrasonic micro-probe was able to precisely determine the location, size and range of the rectal carcinoid tumor, which may guide endoscopic treatment. For lesions <1 cm with clear border, we believe that endoscopic resection, particularly EMR, should be considered first. Pathological examination is needed for final diagnosis and immunohistochemical examination is necessary if the lesion is considered malignant to improve the preoperative diagnosis of carcinoid tumor rate. Due to the fact that other tumors in the colon or other parts are concomitant with a high incidence, we should fully evaluate the colon instead of only treating the local lesions.
